# Using cause-effect graphs to elicit expert knowledge for cross-impact balance analysis

**DOI:** 10.1016/j.mex.2021.101492

**Published:** 2021-08-17

**Authors:** Ivana Stankov, Andres F. Useche, Jose D. Meisel, Felipe Montes, Lidia MO. Morais, Amelia AL. Friche, Brent A. Langellier, Peter Hovmand, Olga L. Sarmiento, Ross A. Hammond, Ana V. Diez Roux

**Affiliations:** a Urban Health Collaborative, Dornsife School of Public Health, Drexel University, 3600 Market St, Philadelphia, PA 19104, USA; b South Australian Health and Medical Research Institute, North Terrace, Adelaide, SA 5000, Australia; c Department of Industrial Engineering, Universidad de Los Andes, Bogotà, Colombia; d Social and Health Complexity Center, Universidad de Los Andes, Bogotà, Colombia; e Facultad de Ingeniería, Universidad de Ibagué, Carrera 22 Calle 67, Ibagué 730001, Colombia; f Observatory for Urban Health in Belo Horizonte, Belo Horizonte, Brazil; g School of Medicine, Federal University of Minas Gerais, Belo Horizonte, Brazil; h Department of Health Management and Policy, Dornsife School of Public Health, Drexel University, 3215 Market St, Philadelphia, PA 19104, USA; i Center for Community Health Integration, Case Western Reserve University, Cleveland, OH, USA; j Department of Public Health, School of Medicine, Universidad de los Andes, Bogotà, Colombia; k Brown School at Washington University in St. Louis, One Brookings Drive, St Louis, MO 36130, USA; l Center on Social Dynamics and Policy, The Brookings Institution, 1775 Massachusetts Ave NW, Washington, DC 20036, USA; m Santa Fe Institute, 1399 Hyde Park Rd, Santa Fe, NM 87501, USA

**Keywords:** Complex Systems, Systems thinking, Scenario analysis, Epidemiology, Urban Health, Chronic disease, Food environment, Diet, Transportation system

## Abstract

Cross-impact balance (CIB) analysis leverages expert knowledge pertaining to the nature and strength of relationships between components of a system to identify the most plausible future ‘scenarios’ of the system. These scenarios, also referred to as ‘storylines’, provide qualitative insights into how the state of one factor can either promote or restrict the future state of one or multiple other factors in the system. This paper presents a novel, visually oriented questionnaire developed to elicit expert knowledge about the relationships between key factors in a system, for the purpose of CIB analysis. The questionnaire requires experts to make selections from a series of standardized cause-effect graphical profiles that depict a range of linear and non-linear relationships between factor pairs. The questionnaire and the process of translating the graphical selections into data that can be used for CIB analysis is described using an applied example which focuses on urban health in Latin American cities.

## Method details

Cross-Impact Analysis methods are a set of methods developed in the 1970s and early 80s. These methods explore the possible futures of a system by accounting for the possibility that the occurrence of a given event, or events, could modify the likelihood that other events will occur [1]. Cross-Impact Analysis methods require experts to provide information about the conditional and joint probabilities of events or factors in a complex system. This presents a significant challenge given that experts “not only need to know which interrelations exist in a system but they also have to recognize which results this impact network will produce” ([3], p.337). Cross-impact balance (CIB) analysis, the focus of this paper, is a variant of these methods which was developed to minimize the amount of mental calculation required by experts. This is achieved by providing a structured means for eliciting expert knowledge about the strength and nature of the relationships between factors in a system. These interrelationships are represented in an impact matrix which characterizes the interaction network of the system, and which can be used to understand how the state of one factor can either promote or restrict the future state of another factor in the system [6]. The CIB algorithm evaluates the impact matrix and identifies the most plausible future configurations or ‘scenarios’ of the system ([3], 2008). These scenarios, also referred to as ‘storylines’, afford a qualitative insight into the system by explicating assumptions about the states of multiple factors in the system [6].

This paper focuses on a novel, visually oriented approach for eliciting the expert knowledge about relationships between key factors in a system that must be collected as part of any CIB analysis. This approach is described by using a case study which applied CIB analysis to examine how the interrelated influence of food and transportation system factors impact future urban health scenarios in Latin American cities [2]. The approach outlined in this paper adds to the CIB methodological toolbox and could be used as a more visual, and simpler, alternative to the standard approach to eliciting judgements from experts. While this paper will describe various aspects of the CIB method, it will not provide a detailed description of the CIB algorithm and how it works. For more information about CIB analysis, readers are directed to the following papers: [3,4].

## Preparation phase

A set of eleven factors were selected for inclusion in the CIB analysis. While there is no limit to the number of factors that can be considered, the size of the CIB matrix significantly increases the time required for experts to evaluate the relationships between factor pairs. The set of factors examined in this paper included factors from the food and transportation system, along with key health and behavioral factors that were perceived to be the most important factors for exploring the future of urban health in Latin American cities. These factors included: (1) prevalence of chronic disease, (2) physical activity, (3) highly processed food consumption, (4) car use, (5) free time, (6) food marketing regulations, (7) sugar sweetened beverage/ processed food taxes, (8) healthy food prices, (9) public transportation subsidies, (10) political will for social change, and (11) street safety.

After deciding on the most important factors, namely those with the most significant direct and or indirect impact on urban health in Latin America, we selected the states for each of these factors. For simplicity, we restricted our focus to two possible states for each factor: ‘low’ and ‘high’. In reality, the states chosen can vary across factors and applications, as their informativeness depends on the focus of the study. For example, it may be more informative to consider three levels of certain factors (e.g., ‘physical activity’ may be categorized as ‘low’, ‘medium’ and ‘high’). In some cases, states can be defined using quantitative cutoffs (e.g., ‘< 5%’ and ‘≥ 5%’ for a factor like ‘public transportation subsidies’). However, this paper focuses on systems where the factors are defined according to just two possible interval states: ‘low’ and ‘high’.

## Traditional approach to eliciting information

To enable the identification of future scenarios, the CIB method relies on experts to evaluate the strength and direction of each pair of factors in the system, in all their possible states. These assessments are traditionally elicited using a focus question. For example, in the context of the factors listed above, the relationship between high car use and low physical activity would be elicited using a focus question of the form ([3], p.339):

If the only piece of information about the system is that *Car use* is *high*, will you evaluate this, due to the direct influence of *Car use* on *Physical activity*, as a hint that *Physical activity* is *low* (promoting influence - positive points) or as a hint that *Physical activity* IS NOT *low* (restricting influence - negative points)?

To answer the question, experts select the most appropriate response from the following options:

+2: strongly promoting direct influence.
+1: weakly promoting direct influence.
 0: no direct influence.
-1: weakly restricting direct influence.
-2: strongly restricting direct influence.

This process of eliciting insights can however, become mentally taxing, particularly when experts are asked about many relationships, requiring responses to nuanced permutations of the focus question. To alleviate some of this mental burden, a visually oriented approach was employed, which included the development of a set of standardized cause-effect profiles (bivariate graphs) from which experts can choose, and a process for transforming these selections into numeric values in the cross-impact matrix which can be analyzed using the CIB algorithm.

## Development of a visually oriented questionnaire

An introductory presentation and an interviewer-assisted questionnaire was developed, which used a combination of questions and visual prompts, in the form of graphs (cause-effect profiles), to elicit participants’ perceptions about the bivariate relationships between system factors and their states. The questionnaire was encoded using an Excel Macro. The introductory presentation and questionnaire were pilot tested on three researchers familiar with systems thinking to help identify any issues relating to the structure of the questionnaire or the instructions provided. The materials were refined based on the feedback received and the introductory presentation and questionnaire were then translated into Spanish and Portuguese. A second round of pilot testing (and revisions) were subsequently undertaken on a further three individuals to ensure the translations were clear and appropriately captured the meaning and intent of the questions.

The introductory presentation was principally used to familiarize experts with the structure of the questionnaire and what they would be asked to focus on. The first part of the presentation reiterated the focus of the questions, including the primary intent of the questionnaire which was to explicate the nature of direct relationships between factor pairs. This focus is fundamental to the CIB method. Thereafter, experts were guided through a set of examples that illustrated how different factors may be related in linear and non-linear ways. The factor pairs used as examples were distinct from the food and transportation system factors that were featured in the questionnaire. For instance, examples included the influence of population size on the count of households in a city, and the relationship between household income and happiness. Importantly, the selection of these examples was informed by the background knowledge of the participating experts, and the issues that were most likely to resonate with them. Please see Supplement material for the introductory presentation.

**Fig. 1:**
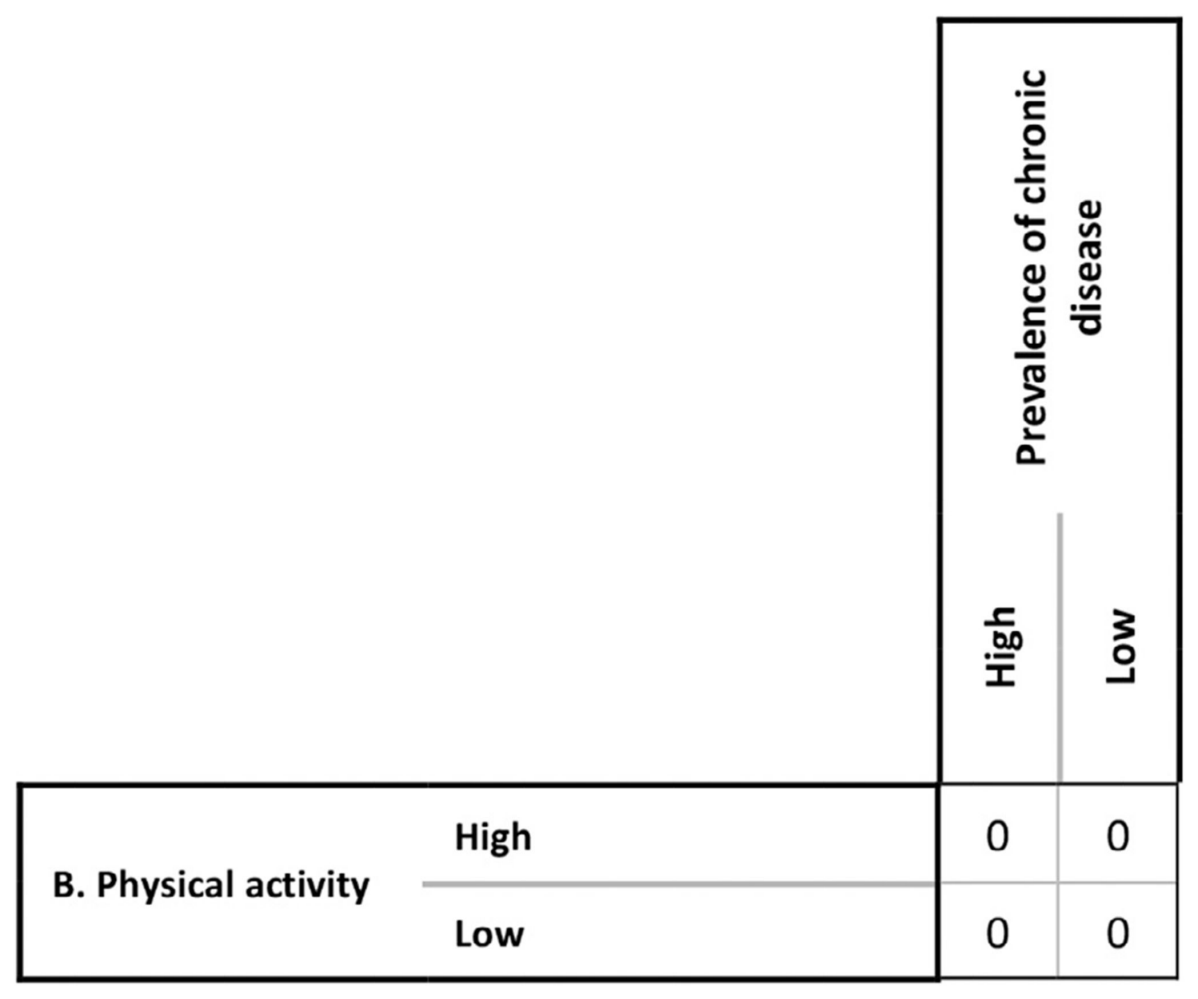
The values entered in the CIB matrix if no direct relationship between physical activity and chronic disease prevalence is identified.

The questionnaire was set up in two parts; (1) the core question, and (2) the selection of the most appropriate cause-effect profile or bivariate relationship between a given pair of factors. The core question was designed to filter out only those factor pairs for which a direct relationship exists. As such, experts were first asked a question in the form: ‘Does factor *X* directly influence factor *Y*’. For example, ‘Does physical activity have a direct influence on chronic disease prevalence?’. Experts with a deeper understanding of systems and causal thinking and causal loop diagramming were further prompted by asking: ‘Could you draw a direct arrow from physical activity to prevalence of chronic disease in a causal loop diagram?’ If the answer was ‘no’ then a set of zeroes were entered into the corresponding region of the CIB matrix and they were asked about the relationship between the next pair of factors (Fig. 1).

**Fig. 2:**
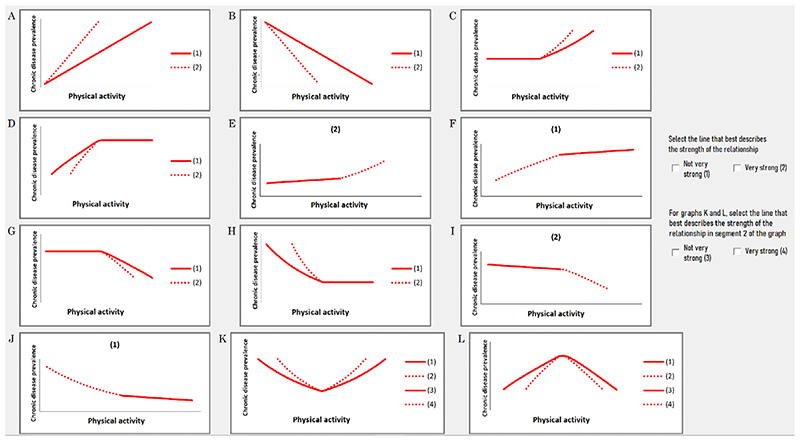
List of all possible bivariate relationships from which experts selected the relationship they perceived best described the influence of one factor on another.

In contrast, if the expert reported that ‘yes’ there was a direct relationship between the two factors (*e.g*., that physical activity does directly influence chronic disease prevalence), then they were asked to select the graph that they thought best represented this relationship (Fig. 2).

The graphs include both linear and non-linear cause-effect profiles and were designed to capture every possible permutation of relationship strengths and profiles between factor pairs and their states (Fig. 2). Experts were encouraged to talk through their thinking and explain their selections. As they made their selections, the nature of the relationship embodied by the chosen graph, in the context of any given factor pair, was verbalized using prompts like: “Let me make sure I understand what you mean. What you’re saying is that: At HIGH levels of *[factor x], [factor x]* has a very strong/ not very strong (promoting/ restricting) influence on *[state high/low* of *factor y] and at* LOW levels of *[factor x], [factor x]* has a very strong/ not very strong (promoting/ restricting) influence on *[state high/low* of *factor y].”* This process played an important role in helping experts clarify the reasoning behind their selections. Importantly, it also afforded an opportunity for experts to recognize when a particular graph selection did not align with their thinking and offered the opportunity to choose a different graph.

**Fig. 3:**
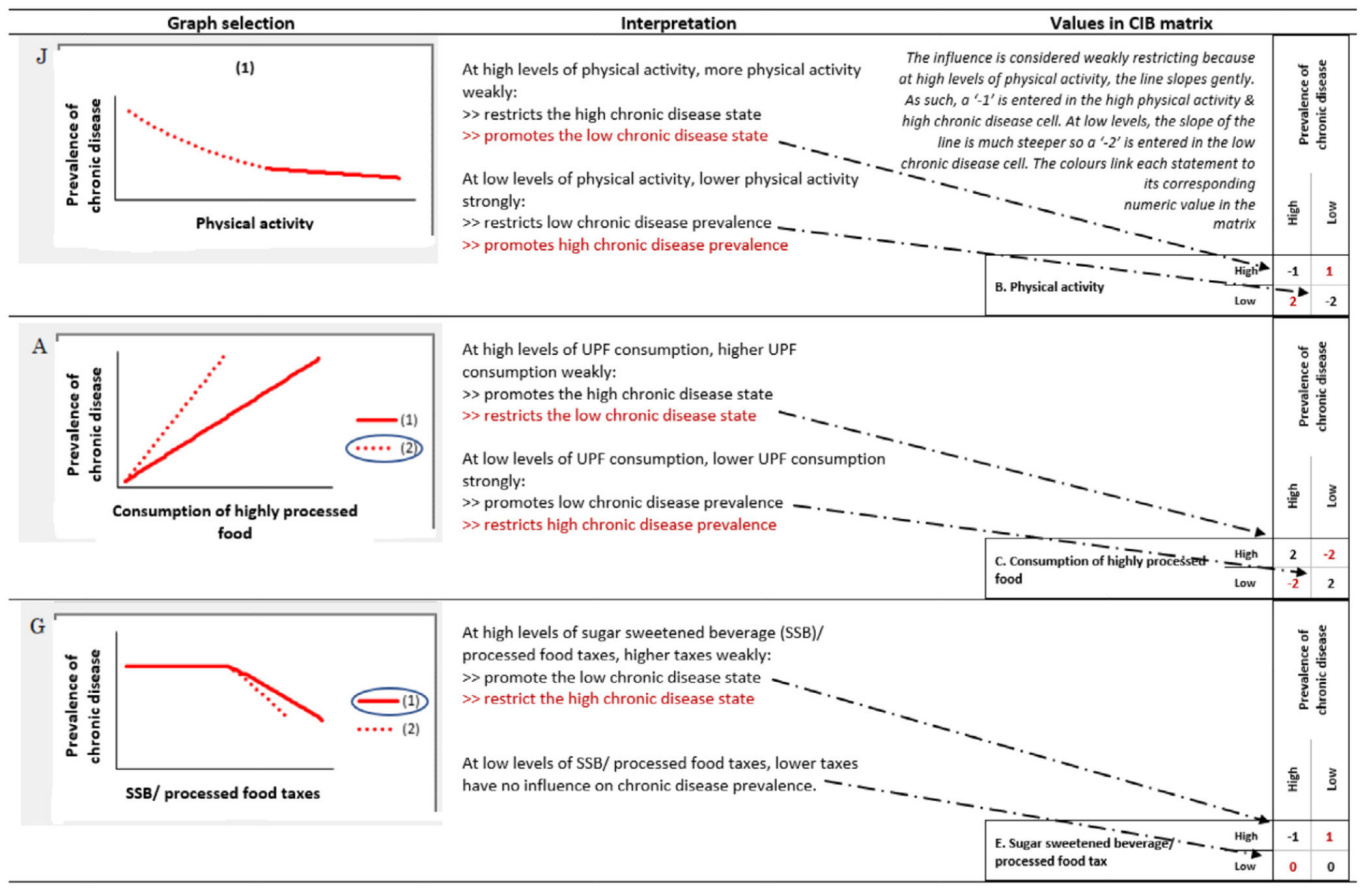
Three examples showing how the graphs selected by experts are interpreted and encoded in numerical form in the CIB matrix.

Outlined in Fig. 3, are three examples of how the graphs were interpreted and verbalized back to experts, and subsequently translated into a numeric code featured in the CIB matrix. An exhaustive list of the numeric codes that correspond to each graph selection are listed in Table 1.

A notable advantage of asking experts to select from a set of possible cause-effect graphs is that experts can rate the influence of the x-axis factor in both states *i.e*., both the ‘low’ and the ‘high’ state, on the *y*-axis factor with just one graph selection, whereas two separate ratings would be required to do this using the traditional approach. Another potential advantage of using the graphs is that they can assist in the identification of non-linear relationships by making them visually explicit. Without a visual prompt, experts may otherwise be tempted to rate the influence of a given factor in a ‘high’ state as the inverse of the influence of that same factor in the ‘low’ state. For example, an expert may deem that high car use strongly promotes low physical activity (resulting in a +2 rating). This determination, in turn, may lead experts to rate low car use as having a strongly restricting influence on low physical activity (*i.e*., a -2 rating). This implies a linear decreasing relationship between car use and physical activity. While such a relationship is completely plausible, the lack of visual cues and the difficulties inherent in interpreting the meaning of complex questions (such as those associated with the traditional approach), may make experts less likely to identify non-linear forms of decreasing relationships, such as those depicted in Fig. 1, graph J(1) or G(2).

Once all ratings were collected and the interaction network explicated, ScenarioWizard v.4.31 was used to conduct the CIB analysis [5] using the usual process [3]. The ScenarioWizard software and its accompanying instruction manual are available as free online downloads for Windows systems. The software can also be operated using Mac OS systems using Windows virtualization tools such as Oracle VirtualBox [5].

## Supplementary Material

Supplementary File 1

## Figures and Tables

**Table 1:** Graph selection and corresponding values in the CIB matrix.

		CIB MATRIX	Target of influence (y-axis factor)		
Graph selection graph(line)		Source of influence		H	L
A(1)	→	**x-axis factor**	**H**	1	-1
			**L**	-1	1
A(2)	→	**x-axis factor**	**H**	2	-2
			**L**	-2	2
B(1)	→	**x-axis factor**	**H**	-1	1
			**L**	1	-1
B(2)	→	**x-axis factor**	**H**	-2	2
			**L**	2	-2
C(1)	→	**x-axis factor**	**H**	1	-1
			**L**	0	0
C(2)	→	**x-axis factor**	**H**	2	-2
			**L**	0	0
D(1)	→	**x-axis factor**	**H**	0	0
			**L**	-1	1
D(2)	→	**x-axis factor**	**H**	0	0
			**L**	-2	2
E(2)	→	**x-axis factor**	**H**	2	-2
			**L**	-1	1
F(1)	→	**x-axis factor**	**H**	1	-1
			**L**	-2	2
G(1)	→	**x-axis factor**	**H**	-1	1
			**L**	0	0
G(2)	→	**x-axis factor**	**H**	-2	2
			**L**	0	0
H(1)	→	**x-axis factor**	**H**	0	0
			**L**	1	-1
H(2)	→	**x-axis factor**	**H**	0	0
			**L**	2	-2
I(2)	→	**x-axis factor**	**H**	-2	2
			**L**	1	-1
J(1)	→	**x-axis factor**	**H**	-1	1
			**L**	2	-2
K(1,3)	→	**x-axis factor**	**H**	1	-1
			**L**	1	-1
K(1,4)	→	**x-axis factor**	**H**	2	-2
			**L**	1	-1
K(2,3)	→	**x-axis factor**	**H**	1	-1
			**L**	2	-2
K(2,4)	→	**x-axis factor**	**H**	2	-2
			**L**	2	-2
L(1,3)	→	**x-axis factor**	**H**	-1	1
			**L**	-2	1
L(1,4)	→	**x-axis factor**	**H**	-2	2
			**L**	-1	1
L(2,3)	→	**x-axis factor**	**H**	-1	1
			**L**	-2	2
L(2,4)	→	**x-axis factor**	**H**	-2	2
			**L**	-2	2

H: high state, L: low state

## Box

- A questionnaire featuring a set of standardized cause-effect profiles was developed.
- Cause-effect profiles were used to elicit information about the strength of linear and non-linear bivariate relationships.
- The questionnaire represents an intuitive visual means for collecting data required for the conduct of CIB analysis.

## Box

**Table:** Specifications table

Subject Area:	Environmental Science
More specific subject area:	*Complex Systems*
Method name:	Cross-impact balance (CIB) analysis
Name and reference of original method:	Weimer-Jehle, W. (2006). Cross-impact balances: A system-theoretical approach to cross-impact analysis. *Technological Forecasting and Social Change*, 73, 334-361.
Resource availability:	Not applicable
