# Supplementary material for: Using cause-effect graphs to elicit expert knowledge for cross-impact balance analysis
Source: MethodsX. 2021 Aug 17;8:101492. doi: 10.1016/j.mex.2021.101492 (PMC7611690; doi:10.1016/j.mex.2021.101492)

DEEPENING UNDERSTANDING OF FOOD AND TRANSPORT SYSTEMS AND THEIR INFLUENCE ON HEALTH

A STUDY COMPARING THREE REGIONS IN LATIN AMERICA

QUESTIONNAIRE STRUCTURE

direct influence ?

factor x factor y

direct influence ?

factor x factor y

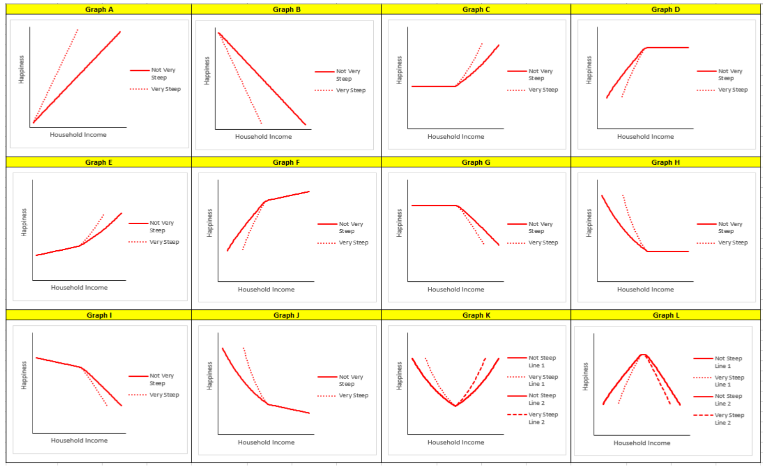

EXAMPLE 1: POPULATION SIZE & HOUSING

The influence of a city's population on the number of houses

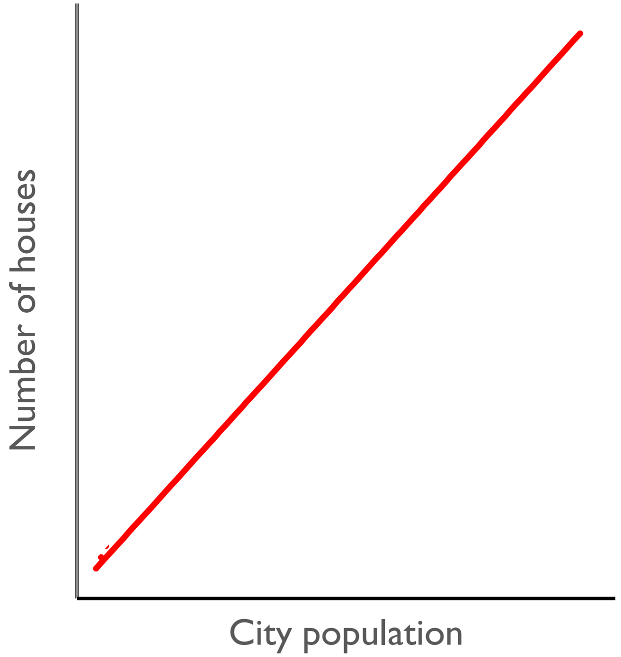

EXAMPLE 1: POPULATION SIZE & HOUSING

The influence of a city's population on the number of houses

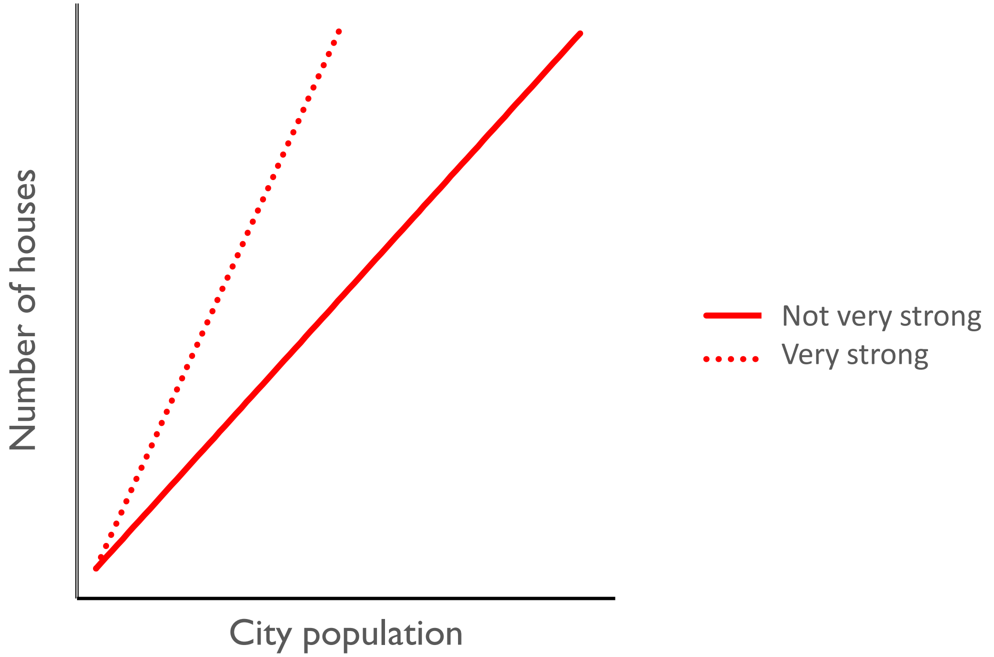

EXAMPLE 2: DISTANCE & PERCEIVED HEAT INTENSITY

The influence of proximity to the heat intensity felt from a stove

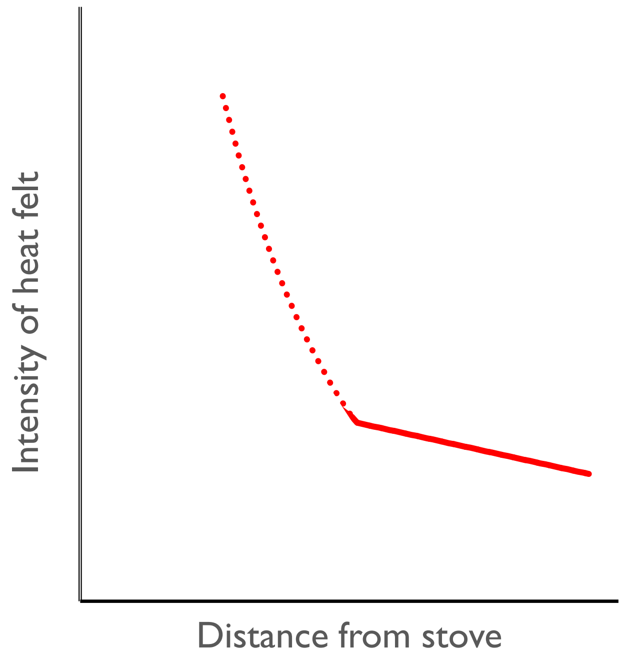

EXAMPLE 3: ALCOHOL CONSUMPTION & DEPRESSION

The influence of alcohol consumption on depressive symptoms

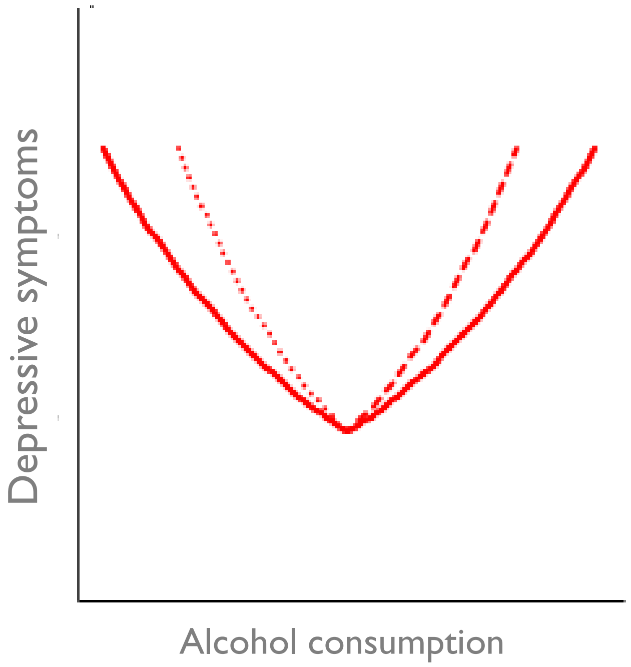

EXAMPLE 4: HOUSEHOLD INCOME & HAPPINESS

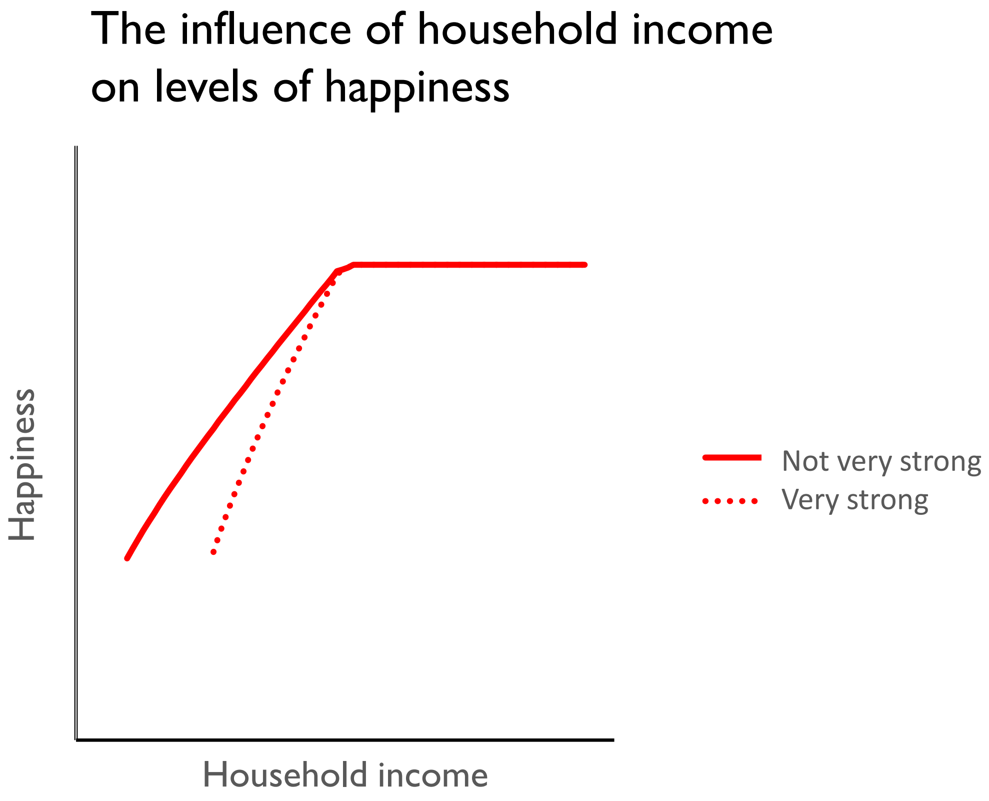

Graph A

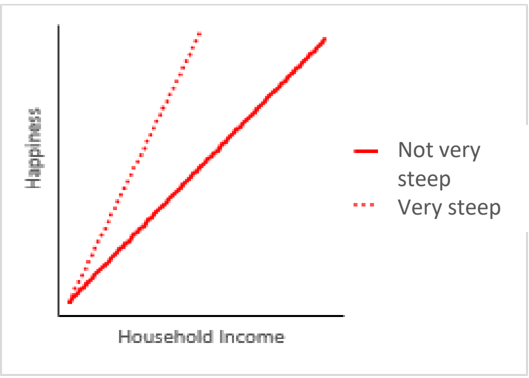

Graph B

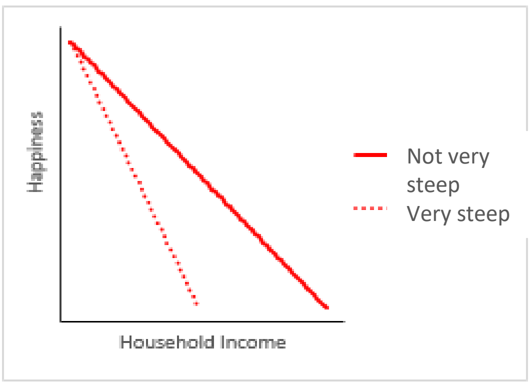

Graph C

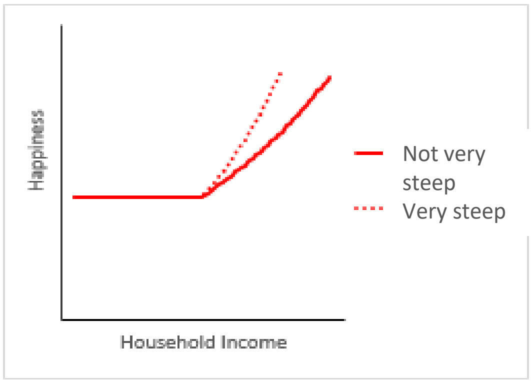

Graph D

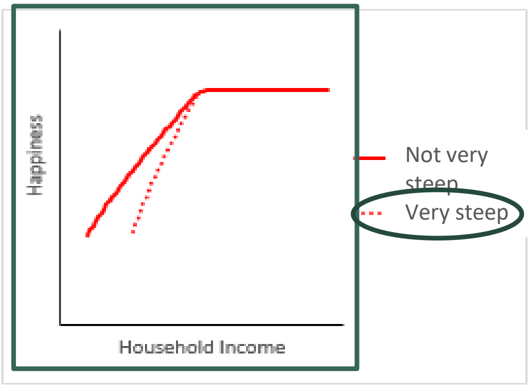

Graph E

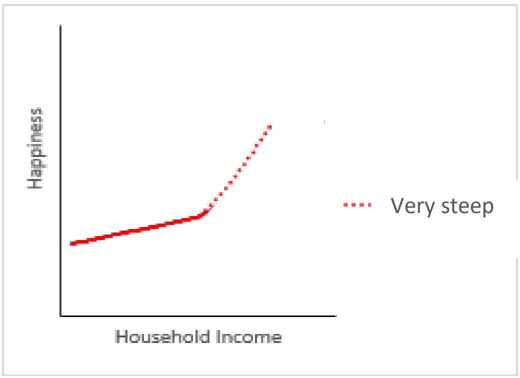

Graph F

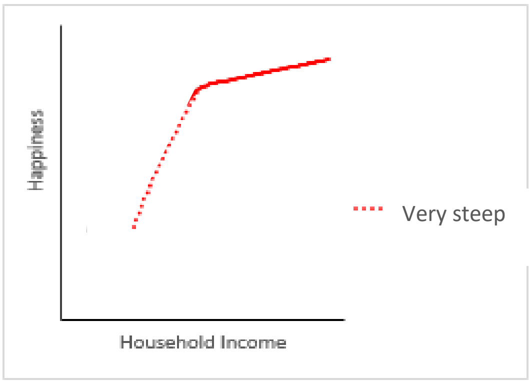

Graph G

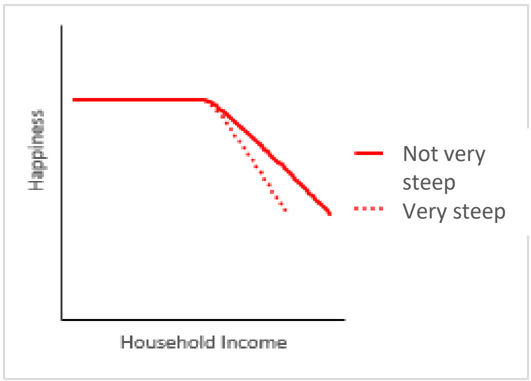

Graph H

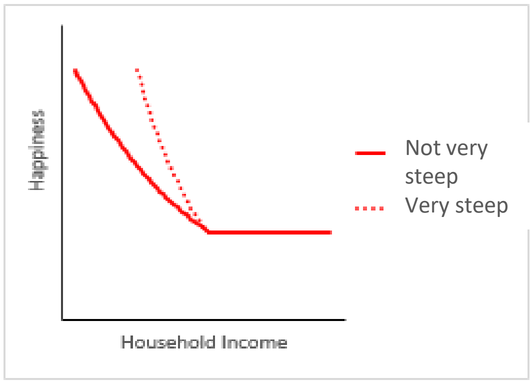

Graph I

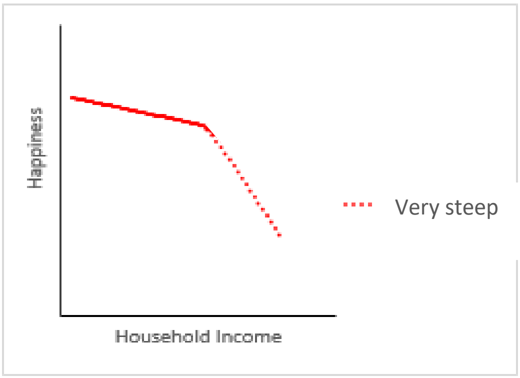

Graph J

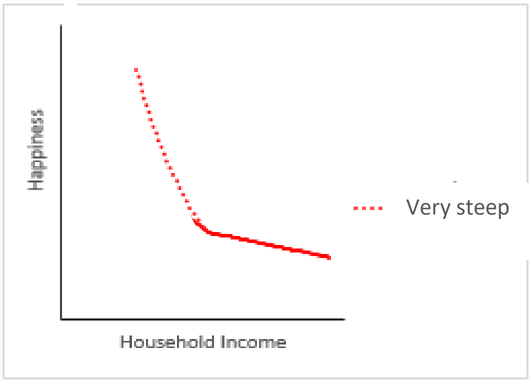

Graph K

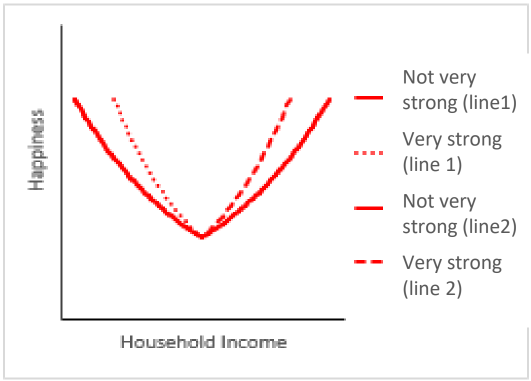

Graph L

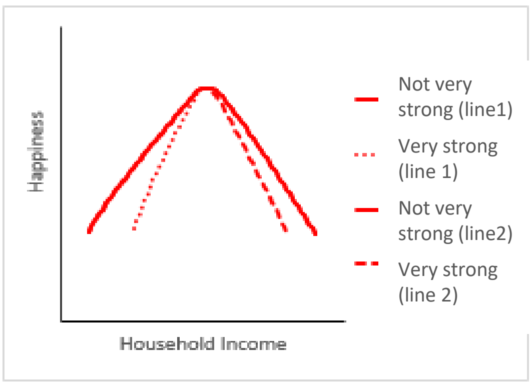

Supplement: Supplementary file 1 [file mmc1.pdf]
